# The Burden Heritage

**Published:** 1977

**Authors:** D. A. Primrose

**Affiliations:** Physician Superintendent Royal Scottish National Hospital, Larbert, Stirlingshire


### The Burden Heritage

**Dr. D. A. Primrose***

Physician Superintendent

Royal Scottish National Hospital, Larbert, Stirlingshire

Progress in humanity is stimulated by individuals, however much organised society takes over the responsibility afterwards. In this respect Harold Nelson Burden was a powerful individual in establishing in this country the official acceptance of responsibilty for the care of mental defectives. He was a member of the 1904 Royal Commission on the Care and Control of the feeble-minded, and out of their Report came the 1913 Mental Deficiency and Lunacy Act. However his interest in mental deficiency started long before his appointment to the Royal Commission. He was well aware from his work in the slums of the East End of London how much poverty and deprivation made worse the conditions of those who are less fitted to compete for the basic necessities of life. One measure of a civilisation is the proportion of its resources which it is prepared to allocate to the sick and the poor. Our Western civilisation is founded on Christianity and this religion has since its inception acted as a curb on man's greed and self-interest and encouraged him to accept responsibility as his brother's keeper. The Church has always taken a leading part in the succour of the sick and the poor and this was one of the functions of the monasteries. On the dissolution of the monasteries some, like St. Mary's of Bethlehem in London, became asylums, and helped to care for the mental defective as well as those whose minds were deranged.

In the 19th century there was a growing realisation that idiots were not lunatics and that with training some of those classified as idiots could be helped to become useful members of society. To begin with, this training was concentrated on children, and the Royal Scottish National hospital where I now work was founded in 1862 as a Training School for the Imbecile Youth of Scotland. Unfortunately children had to leave there on reaching the age of 18 years and one frequently finds reference in the minutes to the need for provision for older mental defectives. For example in the Report by the Medical Officer dated 31st January 1906 there is expressed "the hope that the Royal Commission, which is at present sitting, will provide a remedy for what is equally a disgrace to our civilisation and our Christianity". He was referring to the necessity to discharge the children, once they grew up, into the community with no law to guard them from exploitation and provide for their care. However Mr. Burden did not wait for Government action and in 1902 he founded the National Institution for Persons Requiring Care and Control. As you know this led to th[illegible] purchase of property and in 1909 to the establishmen[illegible] of Stoke Park Colony which then became the firs[illegible] Institution in England to be registered under the 191[illegible] Act.

Our own foundation, although somewhat earlier, wa[illegible] not dissimilar. About 1849, the year in which Esse[illegible] Hall was founded in Colchester, Dr. John Coldstrea[illegible] — who I think had been a medical missionary i[illegible] China — became interested in the work of Dr. Gug genbuhl in Switzerland and from then on he urge[illegible] forward, in Scotland, the cause for the care an[illegible] training of mental defectives. This resulted in sma[illegible] beginnings with firstly a private establishment in Dun dee in 1853 and then a house in Edinburgh in 185[illegible] and seven years later to the opening of an institutio[illegible] in Central Scotland at Larbert to serve all Scotland[illegible] This institution was originally planned to take 20[illegible] children and now has over 1,200 patients of all ages[illegible]

We have not been so fortunate as Stoke Park i[illegible] that our situation mid-way between Glasgow and Edin burgh meant we had little contact with their medica[illegible] schools, and there was no research endowment an[illegible] so we cannot lay claim to such eminent names a[illegible] Professor Berry, Dr. Norman, Fraser-Roberts, Gre[illegible] Walter and Ruth Griffiths — to mention a few — but eminent people like them are a stimulus to stu dents and I remember as a student listening to Gre[illegible] Walter — and also watching Mr. McKissock, who wa[illegible] a neuro-surgeon here, doing brain surgery. Such peopl[illegible] can have great influence on our lives and have n[illegible] doubt had some effect on my interest in the causes o[illegible] mental defect. Perhaps there have been other in fluences of which I was not aware, for about the tim[illegible] when Mr. Burden was working with the poor in th[illegible] East End of London — so also was my grandfathe[illegible] who gave up his work as a doctor in Glasgow to b[illegible] ordained in the Ministry and for some of his time be fore returning to Scotland, he did mission work i[illegible] the London slums. Their paths may well have crosse[illegible] and so I in my turn come here to pay homage to th[illegible] memory of Mr. Burden and the wonderful work whic[illegible] he established at Stoke Park.

* Text of Dr. Primrose's address of thanks on bein[illegible] presented with the Burden Research Medal and Priz[illegible] for 1976, on Thursday, June 10 at Stoke Park Hospi tal.

